# Re-testing of predictive biomarkers on surgical breast cancer specimens is clinically relevant

**DOI:** 10.1007/s10549-018-05119-2

**Published:** 2019-01-18

**Authors:** Stephanie Robertson, Caroline Rönnlund, Jana de Boniface, Johan Hartman

**Affiliations:** 1 Department of Oncology and Pathology, CCK, Karolinska Institutet, 17176 Stockholm, Sweden; 2 Department of Clinical Pathology and Cytology, Karolinska University Laboratory, Stockholm, Sweden; 3 Department of Molecular Medicine and Surgery, Karolinska Institutet, Stockholm, Sweden; 4 Capio St Göran’s Hospital, Stockholm, Sweden; 5 Stockholm South General Hospital, Stockholm, Sweden

**Keywords:** Breast cancer, Core biopsy, Predictive biomarker, Immunohistochemistry, Human epidermal growth factor receptor 2, Ki67

## Abstract

**Purpose:**

The accuracy of predictive and prognostic biomarker assessment in breast cancer is paramount since these guide therapy decisions. The aim was to investigate the concordance of biomarkers and immunohistochemical (IHC)-based surrogate tumor subtypes between core needle biopsies (CNB) and consecutive paired breast cancer surgical resections.

**Methods:**

This retrospective study comprised two cohorts of patients with primary breast cancer diagnosed between 2016 and 2017: one treated with primary surgery (*n* = 526) and one with neoadjuvant chemotherapy (NAC) (*n* = 216). The agreement between preoperative CNB and paired tumor specimens regarding the assessment of biomarkers and surrogate tumor subtypes was evaluated in both cohorts.

**Results:**

In the primary surgery cohort, the concordance rates and kappa values for estrogen receptor (ER), progesterone receptor (PR) and Ki67 were 98.6% (*κ* = 0.917), 89.3% (*κ* = 0.725) and 78.8% (*κ* = 0.529), respectively. Importantly, human epidermal growth factor receptor 2 (HER2) IHC assessment showed only moderate agreement (*κ* = 0.462). HER2 status combining IHC and in situ hybridization was discordant in 3.6% of cases, potentially impacting on indications for HER2-targeted therapy. The concordance rate for IHC-based surrogate tumor subtypes was only 73.2–78.3%. Generally lower concordance rates for ER, PR and HER2 were observed in the NAC cohort. Here, HER2 status was discordant in 7.4%.

**Conclusions:**

The agreement of HER2 and Ki67 between CNB and paired surgical specimen in primary breast cancer is insufficient. Limited agreement of surrogate tumor subtypes indicates a significant clinical value of biomarker re-testing on surgical specimens.

## Introduction

Breast cancer is a heterogeneous disease consisting of several distinct molecular tumor subtypes significantly differing in prognosis and therapeutic response, namely luminal A, luminal B, HER2-enriched and basal-like [1–4]. These intrinsic subtypes can be recapitulated using immunohistochemical (IHC) assessment of the therapy-predictive biomarkers estrogen receptor alpha (ER), progesterone receptor (PR), human epidermal growth factor receptor 2 (HER2) and Ki67 [5–8]. For confirmation of HER2 gene amplification, in situ hybridization (ISH) analysis is performed in tumors equivocal by IHC [9]. International guidelines recommend biomarker assessment to be performed on either core needle biopsies (CNB) or surgical specimens [6, 10, 11]. If IHC is performed on CNB but not on the paired surgical specimen, it could potentially lead to a faulty treatment assignment since therapy-predictive information varies between types of specimen [7, 12].

Among hormone receptor-positive/HER2-negative tumors, high proliferation (high Ki67 expression) distinguishes the more aggressive luminal B-like tumors from the luminal A-like and is therefore clinically important for the decision on neoadjuvant or adjuvant chemotherapy [13–15].

Approximately 15% of all primary invasive breast cancers are HER2 positive, and the accuracy of HER2 analysis is vital. HER2 status is instrumental to select patients with HER2-positive disease for effective targeted anti-HER2 therapy in addition to systemic chemotherapy, without which prognosis is poor [16, 17]. Insufficient concordance of HER2 status between preoperative CNB and surgical specimens can thus dramatically impact on treatment choice [12]. In the setting of neoadjuvant chemotherapy (NAC), some studies indicate insufficient concordance rates for HER2 between pre-NAC CNB and post-NAC surgical specimens, which indicates that HER2 should be re-tested after NAC [18, 19]. The loss of HER2 positivity in the surgical specimen after NAC is associated with poor recurrence-free or disease-free survival and could potentially indicate the need for further systemic therapy [18, 20, 21]. Data on the conversion of biomarkers such as HER2 among Swedish breast cancer patients are largely lacking but an evaluation is highly relevant since laboratory methods can vary between countries and routines for re-testing differ.

Thus, the aim of this study was to investigate the value of re-testing predictive biomarkers in preoperative CNB and paired surgical specimens with a special focus on HER2 and IHC-based surrogate tumor subtypes. In addition, we aimed to investigate the clinical relevance of re-testing biomarkers in patients treated with NAC.

## Materials and methods

### Study cohort

The retrospective study cohort comprised patients with primary invasive breast cancer diagnosed at the Department of Clinical Pathology and Cytology, Karolinska University Laboratory, Stockholm, Sweden, during 2016 and 2017. By specific search criteria in the pathology laboratory information system, we identified 716 cases with data on biomarker assessment both on CNBs and paired surgical specimens. Two cohorts were then created: the primary surgery cohort comprised 526 cases without NAC, and the NAC cohort included 190 patients who had received NAC based on biomarker analyses from CNBs. Cases with complete pathological response (pCR) after NAC, i.e., that had no residual tumor for biomarker assessment left after NAC, or patients treated with neoadjuvant endocrine therapy alone, were not included.

The clinicopathological data retrieved from routine pathology reports comprised tumor characteristics (histological subtype, tumor size, Nottingham Histological Grade), axillary lymph node status, IHC biomarker status for ER, PR, Ki67 and HER2, ISH status for HER2 and tumor response to NAC. Details on type of neoadjuvant therapy were retrieved from the medical record system. The data collection was performed between January 6 and March 17, 2018.

At the accredited laboratory of the Department of Clinical Pathology and Cytology, Karolinska University Laboratory, Stockholm, Sweden, the routine immunohistochemistry staining protocol for breast cancer biomarkers utilized monoclonal rabbit anti-ER (clone SP1), anti-PR (clone 1E2), anti-Ki67 (clone 30-9) and anti-HER-2/*neu* (clone 4B5) antibodies and had been performed according to the manufacturer’s instructions (BenchMark ULTRA Staining Module, Ventana Medical Systems, Arizona, USA). For equivocal HER2 protein expression results by IHC, further analysis with ISH had been performed [16]. Routine biomarker assessment and scoring had been performed according to national guidelines. The reported overall HER2 status was based on combined results from IHC protein expression and gene amplification and included ISH HER2/chromosome 17 probe (C17) ratio and average number of HER2 copy numbers.

### Cutoffs for biomarker concordance analysis

Biomarker values were retrospectively collected, and no new assessments were performed in this study. For biomarker concordance analysis, we applied a cutoff value of ≥ 10% for ER positivity, ≥ 10% for PR positivity and ≥ 20% for high proliferation as measured by Ki67 [10, 22]. Negative HER2 protein expression was defined as score 0 or 1 +, and positive as 3 +. Equivocal tumors, scored 2 +, had in most cases been subjected to additional ISH. After ISH assessment, a HER2 copy/C17 control ratio > 2.0 or an average HER2 copy number > 4.0 signals/cell were classified as HER2 positivity. Negative HER2 status was defined as IHC score 0 or 1 +, or ISH HER2/C17 ratio < 2.0 and HER2 copy number < 4.0 signals/cell [16].

### IHC-based surrogate tumor subtype classification

For subtype classification, we compared two different surrogate definitions: those of the St. Gallen consensus meeting in 2013 and those of the current Swedish guidelines which are based on published work by Maisonneuve et al., see Table 1 [10, 15, 22, 23]. For the latter, we applied Ki67 cutoffs as follows: 0–14% = low, 15–22% = intermediate and 23–100% = high. For ER-positive, HER2-negative tumors with intermediate proliferation, PR levels of ≥ 20% or < 20% divide this group into luminal A-like or luminal B-like, respectively [10, 15].

**Table 1:** Immunohistochemical-based surrogate tumor subtype definitions for breast cancer

Intrinsic surrogate tumor subtype	Clinicopathological surrogate definition
Swedish surrogate tumor subtype	
Luminal A-like	ER positive (≥ 10%) and HER2 negative
	*and*
	Low Ki67 (< 14%)^a^*or*
	Intermediate Ki67 (15–22%) and PR ≥ 20%
Luminal B-like	ER positive (≥ 10%) and HER2 negative
	*and*
	High Ki67 (> 23%) *or*
	Intermediate Ki67 (15–22%) and PR < 20%
HER2-positive/luminal	ER positive (≥ 10%) and HER2 positive
	*and*
	Any Ki67/PR
HER2-positive/non-luminal	HER2 positive and ER negative (< 10%) and PR negative (< 10%)
Triple negative	ER negative (< 10%) and PR negative (< 10%) and HER2 negative
St. Gallen surrogate tumor subtype	
Luminal A-like	ER positive (≥ 1%) and PR positive (≥ 20% [23])
	*and*
	HER2 negative
	*and*
	Ki67 low (< 20%; panel consensus)
Luminal B-like (HER2 negative)	ER positive (≥ 1%)
	HER2 negative
	*and at least one of*
	Ki67 high (≥ 20%; panel consensus)
	PR negative or low (< 20% [23])
Luminal B-like (HER2 positive)	ER positive (≥ 1%)
	HER2 over-expressed or amplified
	*any* Ki67/PR
HER2 positive (non-luminal)	HER2 over-expressed or amplified
	ER and PR absent (< 1%)
Triple negative (ductal)	ER and PR absent (< 1%)
	HER2 negative

*ER* estrogen receptor; *PR* progesterone receptor; *HER2* human epidermal growth factor receptor 2

^a^Ki67 cutoffs are laboratory-specific and provided nationally each year

### Statistical analysis

The Kolmogorov–Smirnov test was used to test for normality, and accordingly only nonparametric tests were applied. Comparison of proportions with categorical outcome was performed with the Chi-square test and Fisher’s exact test, respectively. Nonparametric significance tests for two dependent variables were as follows: Fisher’s exact test for paired samples with two categories and the Marginal Homogeneity test for paired variables with more than two categories (HER2 IHC score and IHC-based subtype). The related-samples Wilcoxon signed rank test was applied for continuous outcome comparisons. Cohen’s κ statistics are presented as a measure of agreement between the biomarker assessments. We applied the Landis and Koch 1977 agreement grades for kappa values: 0.21–0.4 as fair, 0.41–0.6 as moderate, 0.61–0.8 as substantial, and 0.81–1 as almost perfect agreement. In analogy to ‘numbers needed to treat’, we defined ‘numbers needed to re-classify’ (NNRC) as 1/risk of re-classification. The risk of re-classification was calculated as number of cases that changed from positive to negative status or from high to low proliferation, divided by the total number of cases. All statistical tests were two-sided, and significance was considered at *p* < 0.05. All statistical analyses were performed using SPSS version 24.0 (IBM Corp., Armonk, USA). Sankey diagrams illustrating changes in subtype classifications were computed in JSFiddle V.0.5a2 (http://jsfiddle.net).

## Results

### Tumor characteristics and IHC biomarker results

The primary surgery cohort consisted of 526 cases, while the NAC cohort included 190 cases. Tumor and patient characteristics are presented in Table 2. Probably due to the exclusion of pCR cases, axillary lymph node metastases were more frequent in the NAC than in the primary surgery cohort.

**Table 2:** Tumor characteristics for both study cohorts

	Primary surgery cohort (*n* = 526)		Neoadjuvant chemotherapy cohort (*n* = 190)
Diagnostic period	2016–2017		2016–2017
Age at diagnosis, median (years (range))	65 (26–97)		51 (29–85)
Histopathological tumor size, median (mm (range))	20 (1–150)		21 (1–180)

	*n* (%)		*n* (%)
Pathological T-stage (TNM 7)^a^			
pT1	264 (50.2)	ypT1	91 (47.9)
pT2	222 (42.2)	ypT2	72 (37.9)
pT3	39 (7.4)	ypT3	25 (13.2)
Missing	1 (0.2)	Missing	2 (1.1)
Multifocality	103 (19.6)		36 (18.9)
Pathological N-stage (TNM 7)^a^			
pN0	315 (59.9)	ypN0	65 (34.2)
pN1	135 (25.7)	ypN1	86 (45.3)
pN2	44 (8.4)	ypN2	24 (12.6)
pN3	21 (4.0)	ypN3	12 (6.3)
Missing	11 (2.1)	Missing	3 (1.6)
Pathological response to neoadjuvant treatment			
No response	–		21 (11.1)
Partial response	–		146 (76.8)
Response not reported	–		23 (12.1)
Histologic type	Surgical specimen		CNB
Invasive carcinoma NST/ductal	316 (60.1)		130 (68.4)
Invasive lobular carcinoma	99 (18.8)		18 (9.5)
Mucinous carcinoma	15 (2.9)		6 (3.2)
Tubular carcinoma	10 (1.9)		0 (0.0)
Papillary carcinoma	4 (0.8)		1 (0.5)
Mixed subtype	5 (1.0)		0 (0.0)
Unclassified	77 (14.6)		35 (18.4)
Nottingham histological grade	Surgical specimen		CNB
Grade 1	65 (12.4)		7 (3.7)
Grade 2	269 (51.1)		96 (50.5)
Grade 3	189 (35.9)		65 (34.2)
Unclassified	3 (0.6)		22 (11.6)

*CNB* core needle biopsy, *NST* nonspecial type

^a^Pathological T stage for invasive tumor and pathological N stage for regional lymph nodes including sentinel lymph nodes

In the primary surgery cohort, there were significant differences in median ER, PR and Ki67% between CNB and surgical specimen: ER 99% versus 95% (*p* = 0.019), PR 70% versus 60% (*p* = 0.023), Ki67 24% versus 28% (*p* < 0.001), respectively. Overall, 89.2% of the tumors were ER positive and 72.6% PR positive on the surgical specimen. As expected, these figures were somewhat lower in the NAC cohort based on pre-NAC CNB, with 75.8% (*p* < 0.001) and 63.2% (*p* = 0.044), respectively. In agreement with this, 67.5% of tumors in the primary surgery cohort had a high proliferation (Ki67) as compared to 88.4% in the NAC cohort (cutoff ≥ 20%; *p* < 0.001).

Combining HER2 IHC and ISH results into HER2 status, HER2 positivity was 9.5% on CNB and 11.4% on surgical specimens without NAC. In the NAC cohort, HER2 positivity was 17.4% and 12.1%, respectively.

### Concordance of ER, PR and Ki67

In the primary surgery cohort, ER status showed an almost perfect and PR status a substantial agreement (Table 3). For ER, 73 tumors needed to be assessed on CNB in order for one case to be re-classified on the surgical specimen (NNRC = 73), while for PR, NNRC was only 10. Only moderate agreement was observed for Ki67 using a cutoff of ≥ 20% for high proliferation (Table 3). Here, only 5 tumors needed to be assessed on CNB before one was re-classified on the surgical specimen (NNRC = 5). Similar results were seen for ER and PR in the NAC cohort (Table 4). Here, NAC effects showed a decreased proliferative index as measured by Ki67, resulting in an only slight degree of agreement.

**Table 3:** Analysis of agreement between core needle biopsy and surgical specimen for ER, PR and Ki67 in cases treated with surgery as primary therapy

		Surgical specimen		Concordance rate (%)	*κ*-value	NNRC
		ER−	ER+			
	ER−	43	3	98.6	0.917	73
	ER+	4	461			
C		PR−	PR+			
N	PR−	104	31	89.3	0.725	10
B	PR+	22	340			
		Ki67 low	Ki67 high			
	Ki67 low	122	66	78.8	0.529	5
	Ki67 high	44	286			

Positive ER and PR status defined with a ≥ 10% cut off and high Ki67 was defined as ≥ 20%

*CNB* core needle biopsy, *ER* estrogen receptor, *PR* progesterone receptor, *NNRC* numbers needed to re-classify (= 1/(n reclassified/total n))

**Table 4:** Analysis of agreement between core needle biopsy and surgical specimen for ER, PR and Ki67 in cases treated with neoadjuvant chemotherapy

		Surgical specimen		Concordance rate (%)	*κ*-value	NNRC
		ER−	ER+			
	ER−	36	4	96.2	0.887	27
	ER+	3	141			
C		PR−	PR+			
N	PR−	56	6	73.9	0.490	4
B	PR+	41	77			
		Ki67 low	Ki67 high			
	Ki67 low	18	2	40.9	0.075	2
	Ki67 high	108	58			

Positive ER and PR status defined with a ≥ 10% cut off and high Ki67 was defined as ≥ 20%

*CNB* core needle biopsy, *ER* estrogen receptor, *PR* progesterone receptor, *NNRC* numbers needed to re-classify (= 1/(n reclassified/total n))

### Agreement of HER2 status

The agreement of HER2 status between CNB and the surgical specimen could be analyzed in 502 tumors from the primary surgery cohort. HER2 IHC-negative cases were slightly more common in CNB than in the surgical specimen. Concordance of HER2 IHC was only 75.4%, implying a moderate agreement, which clearly improved when combining IHC scores and the subsequent ISH analysis into a negative or positive HER2 status (Table 5). Importantly, 18 tumors (3.6%) had a discordant HER2 status. Out of these, 13 tumors were assessed as HER2 negative on CNB but were positive in the resected specimen. A further 5 tumors were HER2 positive on CNB but turned out negative in the resected specimen. For HER2 status, 28 tumors needed to be assessed on CNB in order for one to be re-classified based on the surgical specimen (NNRC = 28) (Table 5).

**Table 5:** Analysis of agreement between core needle biopsy and surgical specimen for HER2 in cases treated with surgery as primary therapy

		Surgical specimen			Concordance rate (%)	*κ*-value	NNRC
		HER2 IHC neg	HER2 IHC equivocal	HER2 IHC pos			
	HER2 IHC neg^a^	299	70	3			
C	HER2 IHC equivocal	41	51	7	75.4	0.462	–
N	HER2 IHC pos	1	3	34			
B		HER2 neg	HER2 pos				
	HER2 neg^b^	439	13		96.4	0.813	28
	HER2 pos	5	45				

*CNB* core needle biopsy, *HER2* human epidermal growth factor receptor 2, *NNRC* numbers needed to re-classify (= 1/(n reclassified/total n))

^a^HER2 IHC score: 0–1 + = negative, 2 + = equivocal, 3 + = positive

^b^HER2 status based on IHC score and results from in situ hybridization analysis

Among the discordant cases in the primary surgery cohort, 6 equivocal cases (IHC 2+) with identical IHC score between CNB and surgical specimen, showed HER2 amplification only on the surgical specimen. This underlines the value of re-testing HER2 even for equivocal cases with a negative ISH on CNB, as HER2 status guides clinical treatment decisions.

In the NAC cohort, the agreement of HER2 status between pre-NAC CNB and post-NAC histopathology was substantial, but only moderate for HER2 IHC assignment (Table 6). Of clinical interest, 10 tumors were HER2 positive on CNB but lost either HER2 expression and/or gene amplification after NAC. Conversely, one tumor assessed as negative on CNB had a positive HER2 status after NAC.

**Table 6:** Analysis of agreement between core needle biopsy and surgical specimen for HER2 in cases treated with neoadjuvant chemotherapy

		Surgical specimen			Concordance rate (%)	*κ*-value	NNRC
		HER2 IHC neg	HER2 IHC equivocal	HER2 IHC pos			
	HER2 IHC^a^ neg	103	13	1			
C	HER2 IHC equivocal	22	29	1	76.7	0.539	–
N	HER2 IHC pos	3	4	13			
B		HER2 neg	HER2 pos				
	HER2^b^ neg	144	1		93.8	0.757	16
	HER2 pos	10	21				

*CNB* core needle biopsy, *HER2* human epidermal growth factor receptor 2, *NNRC* numbers needed to re-classify (= 1/(n reclassified/total n))

^a^HER2 IHC score: 0–1 + = negative, 2 + = equivocal, 3 + = positive

^b^HER2 status based on IHC score and results from in situ hybridization analysis

### Concordance for IHC-based surrogate tumor subtypes

**Fig. 1:**
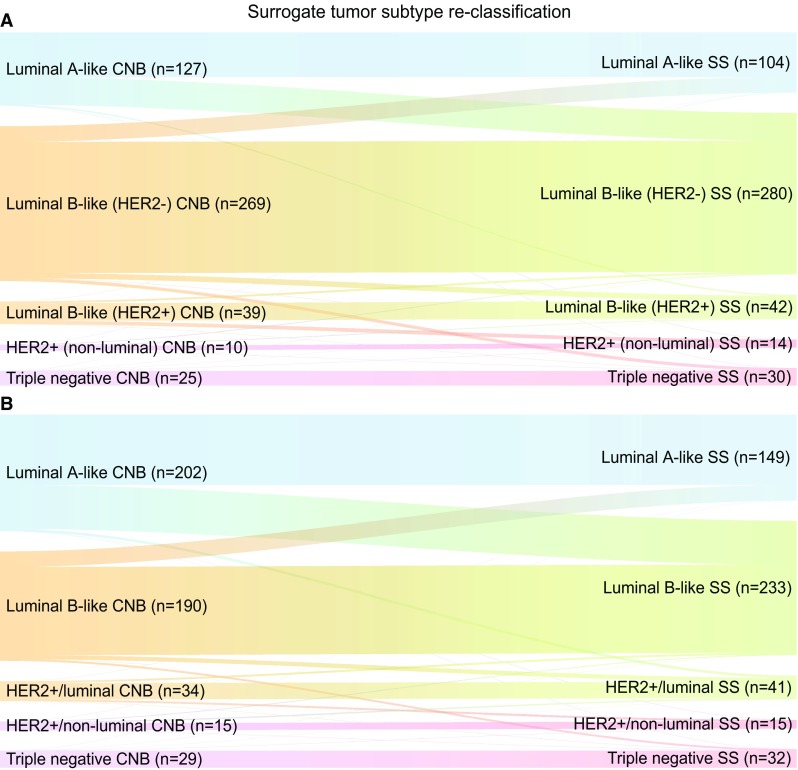
Sankey diagrams for immunohistochemical (IHC)-based surrogate tumor subtype re-classification in core needle biopsy (CNB) versus paired surgical specimen (SS) in the primary surgery cohort. Surrogate tumor subtype re-classification according to St. Gallen definitions (**a**) and Swedish guideline definitions (**b**)

We performed IHC-based surrogate subtype classification on CNB and surgical specimens, both according to the St. Gallen International Expert Consensus from 2013 and the current Swedish guidelines [8, 10]. In the primary surgery cohort, 470 tumors had complete data for comparison of subtype classification according to St. Gallen and Swedish guideline subtypes. The concordance rates applying St. Gallen subtype and the Swedish guideline definitions were 78.3% (*κ* = 0.631) and 73.2% (*κ* = 0.589), respectively. Using Swedish guideline definitions, 76 out of 202 (37.6%) luminal A-like tumors diagnosed on preoperative CNB were re-classified into luminal B-like on the paired surgical specimen. Similar results were observed with the St. Gallen subtype definitions (48 out of 127, 37.8%) (Fig. 1). The overall concordance rate for luminal HER2− tumors only in the primary surgery cohort was 80.3% and 72.8% with St. Gallen and Swedish definitions, respectively.

**Fig. 2:**
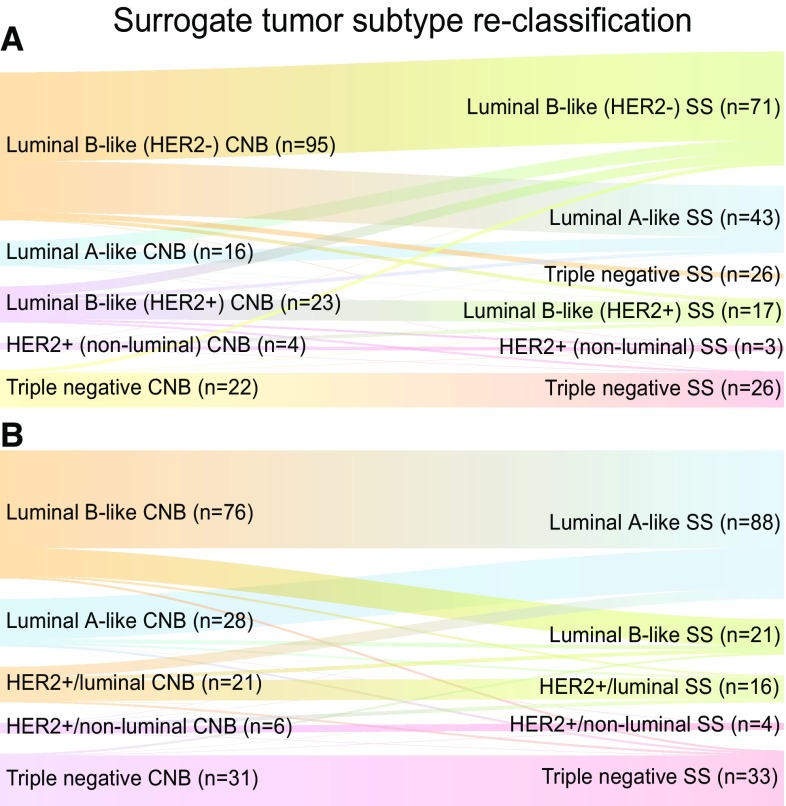
Sankey diagrams for immunohistochemical (IHC)-based surrogate tumor subtype re-classification in core needle biopsy (CNB) versus paired surgical specimen (SS) in the NAC cohort according to definitions by St. Gallen (**a**) and Swedish guidelines (**b**)

Among cases in the NAC cohort, the corresponding concordance rate for all subtypes with St. Gallen definitions was 63.8% (*κ* = 0.460, total *n* = 160) and 53.1% with the Swedish guideline definitions (*κ* = 0.408, total *n* = 162). (Fig. 2).

## Discussion

Core needle biopsy is a well-established and reliable diagnostic method for histopathological diagnosis of breast carcinoma and provides sufficient material for IHC assessment of biomarkers [24, 25]. In addition, the accuracy of biomarkers including ER, PR, HER2 and Ki67 in pre-therapeutic biopsies of early breast cancer is of great importance for therapy decisions and especially for the selection of candidates for NAC. Adjuvant therapy planning may be based on either CNB or surgical specimen, although there are inconsistent results regarding the concordance of specific biomarkers [26–29].

In the present study, we investigated the agreement of biomarkers between CNB and surgical specimen in patients who underwent surgery as primary treatment or received neoadjuvant chemotherapy. The higher concordance rate for ER than PR in this study is in line with previous findings [27, 30]. A previous study by Chen et al. reported approximately 14% re-classification rate from luminal A-like on CNB to luminal B-like on surgical specimens among hormone receptor (HR)+/HER2− tumors, and suggested Ki67 to be repeated on surgical specimen [31]. In the present investigation, the re-classification rate was substantially higher. Since luminal B-like tumors are more likely to receive chemotherapy and to be subject to NAC, this could have significant clinical implications. There is a trend toward an underestimation of the proportion of luminal B-like tumors on CNB. Even though Ki67 is an important factor in distinguishing luminal-like disease, it is prone to inter- and intraobserver variability and has a heterogeneous expression [32, 33]. You et al. reported concordance rates as high as 83.5% (*κ* = 0.647) for Ki67, probably because Ki67 index had been assessed using 10% intervals instead of a continuous percentage (20% cutoff for high proliferation). Furthermore, they reported a HER2 IHC concordance of 84.8% (*κ* = 0.684) [34].

We show a lower overall concordance for IHC-based surrogate tumor subtypes using St. Gallen definitions than previous studies [30, 31]. Meattini et al. showed a significantly higher concordance rate between CNBs and surgical specimens than our study: 87.1% (*κ* = 0.78). Among HR+/HER2−tumors, however, the agreement was lower [30].

Even though the concordance rate for HER2 status including ISH was high, already a small discordance rate may have serious implications for patients not receiving life-saving HER2-targeted therapy. In a large study by Arnedos et al., the reported HER2 concordance rate was as high as 98.8% and showed low levels of amplification in discordant HER2 cases [27]. A high concordance for HER2 (98.3%) was also reported by Lorgis et al., although their cohort comprised a low number of HER2-positive tumors (5.7%), which appears to be insufficient for further conclusions [28]. In a large study with pooled data, Dekker et al. reported a 97.8% concordance for HER2 status [35]. Slightly lower HER2 status concordance rates have also been reported [30, 35]. In the present study, HER2 assessment between 2016 and 2017 followed recent ASCO/CAP guidelines [16]. In the updated guidelines, histopathologic features are suggested for consideration in HER2 discordant cases [9]. Caution should be taken when assessing HER2 in biopsy specimens as they may be affected by artefacts, which can potentially lead to inaccurate HER2 assignment [36, 37].

Breast cancer is a heterogeneous disease exhibiting phenotypic and genetic intra-tumor heterogeneity [38–40]. Studying different areas of the same tumor, biomarker protein (IHC) expression shows larger intra-tumor heterogeneity than observed mRNA levels, especially for Ki67 and PR [41]. Furthermore, significant discordance of HER2 between CNB and surgical resection specimen has been seen among heterogeneous tumors [12]. Sampling different tumor regions as with CNBs may therefore affect biomarker agreement as seen in the present study. Low concordance in biomarker status has also been identified between aspiration cytology and histology-based assessments as a sign of sampling error [42, 43].

Changes in biomarker status after NAC hold important information about tumor biology. Upon assessment, loss of HER2 expression or gene amplification was observed in 10 tumors and conversely, one tumor showed HER2 gene amplification after NAC. Hormone receptor and HER2 status may change during tumor progression [44]. Intrinsic molecular subtypes have been investigated in paired primary and metastatic samples and are generally maintained during metastatic progression. Results from Cevaljo et al. showed, however, that 55% of initially luminal A tumors converted to luminal B and HER2-enriched [45]. The conversion of luminal A primary tumors to non-luminal A metastatic disease has been demonstrated repeatedly [46]. Interestingly, Cevaljo et al. also found an increase in fibroblast growth factor receptor 4 (*FGFR4*) gene expression but not *ERBB2* expression in HER2-enriched metastasis that had converted from luminal primary tumors [45]. Overexpression of *FGFR4* is a characteristic feature of the HER2-enriched subtype.

There are certain limitations to be considered. First, the study had a retrospective design, and results cannot be generalized. Second, a number of patients were diagnosed at other laboratories and referred to the hospital for surgery. CNBs diagnoses were confirmed on surgical resections, but biomarkers and in situ hybridization results were not always re-assessed, and thus not included in the study. Third, patients subjected to CNB instead on fine needle aspiration cytology before surgical treatment are a selected group as they tend to have larger or node-positive tumors prone to receive NAC. It could also be patients with inoperable disease or those not fit for surgery. In addition, patients with complete pathological response to NAC were not included in this study as no remaining tumor tissue could be assessed.

To conclude, the agreement of HER2 and Ki67 between CNB and paired surgical specimen in primary breast cancer is insufficient. An only moderate agreement of surrogate tumor subtypes indicates the clinical value of biomarker re-testing on surgical specimens, and re-testing of at least HER2 and Ki67 should be considered to optimize tailored adjuvant therapy especially for patients treated with NAC.
